# Congenital anomalies observed in children conceived through assisted reproductive technology—a systematic review and meta-analysis

**DOI:** 10.1007/s10815-025-03454-0

**Published:** 2025-03-31

**Authors:** Paripoorna Bhat, Vijay Shree Dhyani, Vani Lakshmi, Shubhashree Uppangala, Satish Kumar Adiga, Prashanth Adiga, Pratap Kumar, Aditi Gupta

**Affiliations:** 1 Division of Reproductive Genetics, Department of Reproductive Science, Kasturba Medical College, Manipal, Manipal Academy of Higher Education, Manipal, Karnataka 576104 India; 2 Kasturba Medical College, Manipal, Manipal Academy of Higher Education, Manipal, Karnataka 576104 India; 3 Department of Data Science, Prasanna School of Public Health, Manipal, Manipal Academy of Higher Education, Manipal, Karnataka 576104 India; 4 Centre of Excellence in Clinical Embryology, Department of Reproductive Science, Kasturba Medical College, Manipal, Manipal Academy of Higher Education, Manipal, Karnataka 576104 India; 5 Department of Reproductive Medicine and Surgery, Kasturba Medical College, Manipal, Manipal Academy of Higher Education, Manipal, Karnataka 576104 India

**Keywords:** Congenital anomalies (CAs), Assisted reproductive technology (ART), In vitro fertilization (IVF), Intracytoplasmic sperm injection (ICSI), Systematic review, Meta-analysis

## Abstract

Congenital anomalies (CAs) are a leading cause of perinatal and child mortality. With the increasing use of assisted reproductive technology (ART), there is a growing need for research on the health outcomes of children conceived through ART. This systematic review was performed to assess the incidence of CAs in ART-conceived children compared to those conceived naturally. This review followed the PRISMA 2020 guidelines and was registered with PROSPERO. A total of 113 studies were included in this meta-analysis, comprising 768,929 children in the ART group and 40,709,337 children in the control group which comprised spontaneously conceived (SC) children. The primary findings indicated that ART-conceived children have a marginally higher risk of CAs compared to the control group. Subgroup analyses showed that children conceived via ICSI, Day 3 transfer, and fresh embryo transfer (ET) had a slightly higher risk of CAs than those conceived via IVF, Day 5 transfer, or frozen embryo transfer (FET). The study highlights a slightly increased incidence of CAs among ART-conceived children over SC children, underscoring the importance of improving ART methods and closely monitoring the health of these children to reduce the risk of CAs.

**Supplementary Information:**

The online version contains supplementary material available at 10.1007/s10815-025-03454-0.

## Introduction

### Background

Congenital anomalies (CAs), more commonly called birth defects, are structural or functional anomalies that develop prior to birth. They include congenital heart defects (CHDs), chromosomal defects such as aneuploidies like Down syndrome, and neural tube defects that lead to poor quality of life and mortality in some cases [1]. Together, they are the primary reason for global perinatal mortality (approximately 2.4 lakh newborns) and global child mortality from ages 1 month to 5 years (equal to around 1.7 lakh children a year). A recent online survey conducted among Southeast Asian countries reported the incidence of birth defects to be 50,000 in 4.6 million children [2]. Chromosomal anomalies like Turner, Edwards, Down, DiGeorge, and Patau syndromes account for 15% of these anomalies, leaving most cases unexplained [3]. CHDs, the most common, occur in 0.76% of live births, rising to 1.5% in stillbirths or terminated pregnancies, highlighting the need for further research [3].

The issues associated with fertility encompass serious social, demographic, as well as medical problems. The World Health Organization (WHO) explains infertility as failure to achieve pregnancy after 12 months or more of regular unprotected sexual intercourse. Roughly one in six people are affected globally with infertility issues. Countries like Poland recommend couples who are trying for conception to directly visit fertility clinics if they are above 40 years of age and to begin with diagnosis and treatment for infertility for couples within 35 years, after unsuccessful attempts [4]. A recent survey-based study in India states that the prevalence of infertility is as high as 24.1 per 1000 women after a duration of marriage of 3 years or more [5]. Assisted reproductive technology (ART) has revolutionized reproductive medicine by aiding conception through in vitro handling and fertilization of gametes. Since the birth of the first in vitro fertilization (IVF) baby in 1978, by performing approximately three million cycles per year, greater than ten million children are credited to be born via ART to date [6, 7]. The advent of procedures in ART like IVF, intracytoplasmic sperm injection (ICSI), and cryopreservation of embryos and gametes, has revolutionized the ART industry [8]. As a result of transferring fewer embryos, rates of healthy pregnancies have increased over this period of time and multiple pregnancy rates have declined, leading to improved neonatal outcomes in several countries [9]. These procedures have become essential tools for overcoming reproductive challenges. However, the increasing use of ART has brought up questions regarding the health of ART-conceived offspring, particularly regarding the risk of CAs [10]. Previous studies have reported an increased incidence of neurological, respiratory, developmental disorders, and facial deformities in ART-conceived children [8]. CAs have been linked to existing demographic parental factors like age, BMI, infertility conditions, and genetic/health problems. The association between ART and CAs tends to reduce after adjusting these factors [9–12]. Not just ART procedures, but additional interventions like ovulation induction, hormone administration, and other methods are also reported to be added to the risk factors but have not been individually proven as the results available are not consistent [12, 13].

The key focus of this review is to infer from the findings of a wide range of studies that have examined the incidence of CAs in ART-born children compared to spontaneously conceived (SC) children. Even with a heterogeneous collection of studies from various parts of the world, the results reported are inconsistent.

### Rationale

The rationale behind this review arises from this lack of consistency and unavailability of meta-analysis studies that include the Indian cohorts with worldwide studies. The existing literature is filled with controversial research outcomes where some present increased incidence and others present inconclusive or contradictory results. The root cause of this heterogeneity arises from the difference with respect to study design, sample size, the criteria chosen for defining CAs, and so on [14]. Possible contributors to this increased incidence include a varied range of ART procedures, from fertilization methods, and cryotechniques to embryo culture techniques and time of embryo transfer. Also, several reviews fail to take parental demographics and infertility conditions into consideration which generates the need for a comprehensive review. Our study aims to provide recent evidence and examine the relationship between ART and CAs through systematic review/meta-analysis [14].

### Objectives

This systematic review and meta-analysis primarily intend to evaluate the association between CAs and ART. First, we seek to know the incidence of these anomalies in children born through ART as opposed to SC offspring. By conducting a meta-analysis, we aim to provide more concrete and up-to-date evidence from the literature. We also divided the studies before and after 2015 to check if the results vary in recent times as compared to earlier times, due to advancements in ART. Second, we plan to investigate possible connections between patient demographics, such as underlying infertility issues, and congenital defects seen in children conceived with assisted reproductive technology. Finally, we want to evaluate the impact of different types of ART interventions on the probability of congenital abnormalities in progeny. By achieving these goals, the research hopes to provide insightful knowledge on the efficacy and safety of ART, directing clinical procedures and influencing future investigations in this field of reproductive medicine.

## Materials and methods

### Methodology and registration

This review followed the Preferred Reporting Items for Systematic reviews and Meta-Analyses (PRISMA) 2020 guidelines (Supplementary Information I) [15]. This review was registered in PROSPERO on 20/05/2024 (ID: CRD42024548861). A protocol was not published prior.

### Study selection and eligibility criteria

The population, intervention, comparison, outcome (PICO) criteria was chosen for the systematic review. All the English language studies following these criteria were chosen for the analysis: (1) studies which involved children with congenital defects conceived through ART or naturally, (2) studies reporting on children conceived through many techniques involved in ART were assessed individually (IVF, ICSI, type of embryo transfer (fresh ET or frozen ET), and time of transfer (Day 5 or Day 3), (3) studies which compared the prevalence of CAs in children conceived through ART with children conceived naturally, and (4) all cohort studies, case series, retrospective cohort studies, clinical trials, observational studies, comparative studies, and prospective studies which reported the incidence of CAs in children. No timeline was applied to select the publications.

The studies that were excluded were as follows: (1) studies that focused solely on naturally conceived children; (2) studies that did not report CAs but some other outcome; (3) studies that included non-human subjects; (4) reviews, meta-analyses, editorials, letters, case reports, and commentaries; (5) studies which reported less than 100 cases; and (6) studies which reported other ART interventions without the ones opted in inclusion (hormonal therapy, IUI, etc.) [8].

The studies were grouped based on objectives which included (1) reporting the incidence of CAs in the ART group and natural conception group, (2) reporting the comparison in incidence of CAs in children born to infertile and fertile couples undergoing ART, and (3) reporting the comparison in incidence of congenital defects in children born through different types of ART technique.

### Information sources and search strategy

Search was conducted using a combination of key terms and database-specific controlled vocabulary for congenital defects and ART. A comprehensive search was performed in PubMed using keywords and MeSH terms combining them with appropriate Boolean operators and field tags and later adapted for Web of Science, Embase, and Scopus (Supplementary Information II). Only English language articles with human subjects published from database inception till 8th May 2024 were searched. A few studies were also searched manually or taken from references of other studies and reviews to include more relevant studies [3]. All the searches were saved in respective databases and all the studies included were recorded in Zotero [16]*.* The search was conducted by two independent investigators (PB and AG).

### Study screening

**Fig. 1:**
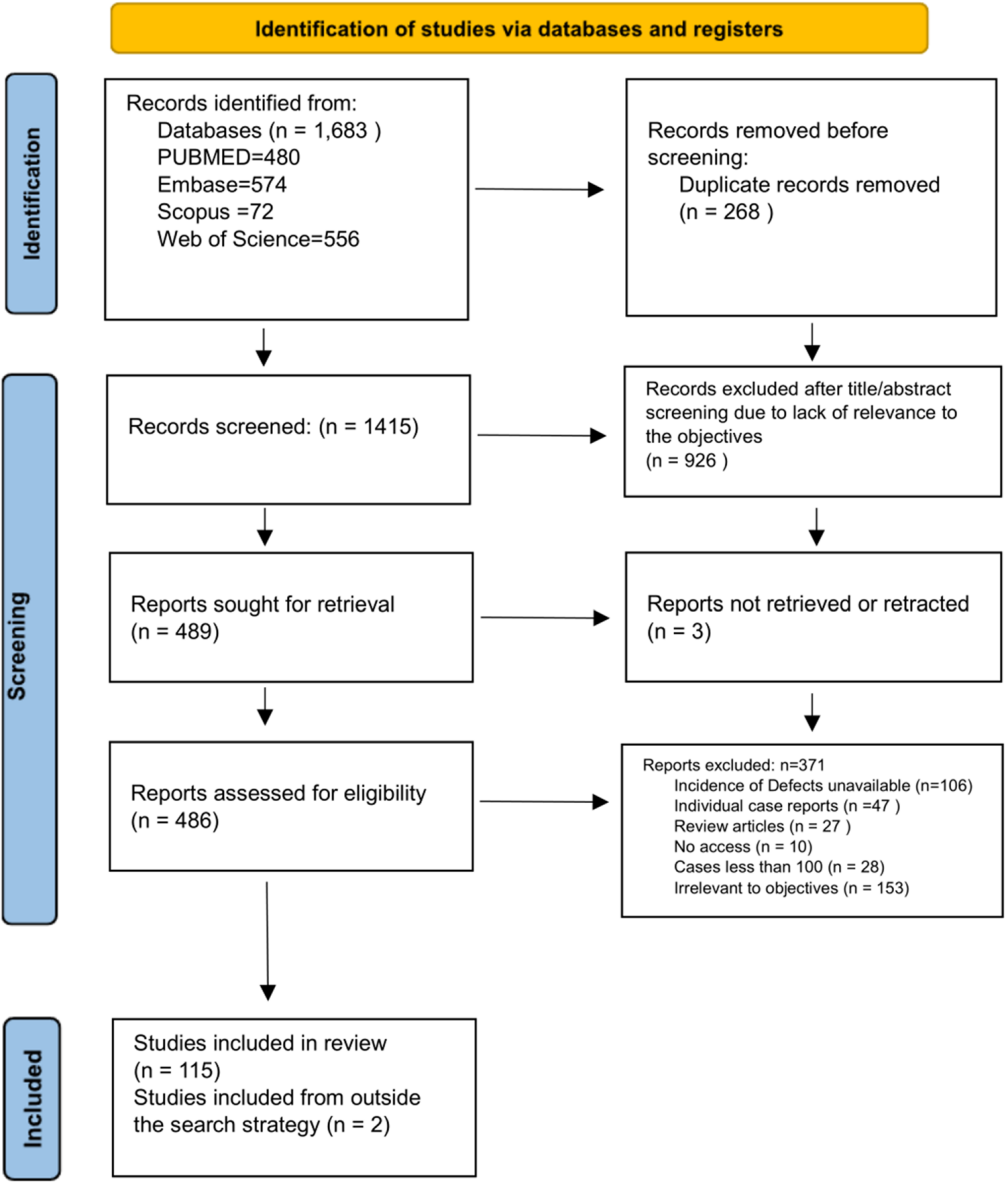
PRISMA 2020 Flowchart for systematic reviews

All the screened articles were reviewed by two investigators (PB and AG) using Rayyan, a software platform that facilitates conducting systematic reviews [17]. Discrepancies raised were resolved through discussion or a third reviewer (SU). Studies overlapping in the objectives were added to both data sheets [18, 19] and duplicates were removed with the help of Rayyan software. The whole process was recorded in PRISMA 2020 flowchart for systematic reviews as given in Fig. 1.

### Data extraction

The data was collected using a predefined data extraction tool designed on Google Sheets (Supplementary Information III). A set of variables were collected for the analysis which are mentioned below. Various publication details like title, journal, country, year, author, methodological details (types of study, population, and sample characteristics), and the outcome data (numbers and percentages) were extracted. The CAs reported with the history of infertility and type of ART intervention were noted. Any additional interventions which did not fall under the intervention technologies we opted (like the use of donor gametes, preimplantation genetic testing, intrafallopian transfers, and any surgical procedures used) were also noted to remove discrepancies.

### Risk of bias assessment

The risk of bias (ROB) was assessed using the case–control studies checklist issued by JBI (The Joanna Briggs Institute) critical appraisal tools [20]. Each study was assessed as per the checklist by two reviewers (PB and AG) independently. The assessment considered the criteria relevant to our systematic review such as study groups, participant identification criteria, statistical analysis, outcome, and other confounding factors (Supplementary Information IV).

### Effect measures and data synthesis

The analysis was conducted in Jamovi software, a free and open statistical platform (https://www.jamovi.org/about.html) [21–23]. The outcome was assessed in a random effect model (DerSimonian-Laird) using log risk ratios. The risk of bias was also assessed using the Fail-Safe N method (Rosenthal) and outliers were identified with the help of a funnel plot. For one sub-analysis, a fixed effect model was used. The primary outcome was to compare the incidence of CAs in ART-conceived children and SC children. This was obtained using a log risk ratio from 70 studies. The study characteristics were described narratively.

For inclusion, homogeneity in the studies such as standardized outcome measurement, hypothesis, and objectives were taken into consideration. A meta-analysis was conducted for 113 out of 117 articles included by computing the risk ratio in Jamovi. I^2^test was used to estimate heterogeneity among the selected studies and funnel plot to assess asymmetry (using rank correlation and regression test). Cook’s distance was used to assess outliers in the study groups. A forest plot was created which displayed the risk ratios and confidence intervals.

## Results

### Study selection

The database search retrieved 1683 articles and after deduplication in Rayyan software, 1415 articles were subjected to title/abstract screening. After removing studies that were irrelevant to the three study objectives, 489 articles were left which were subjected to full-text screening and resulted in 115 articles being included. Additionally, two articles were added from random searches (Fig. 1). From the total 117 studies, 113 were included for analysis since the sample size was not mentioned for the control group for the excluded 4 studies [24–27]. Seventy studies of 113 were included for the meta-analysis performed for objective 1, and 66 studies of 113 for the meta-analysis done for objective 3.

### Characteristics of included studies

To remove potential bias by region, countries from each study were noted. Based on the continental distribution, the maximum studies were obtained from Asia, followed by Europe (Table 1).

**Table 1:** Geographical distribution of studies

Region	No. of studies (References)
Asia	46 [12, 25, 26, 28–70]
Europe	42 [18, 24, 27, 71–109]
Australia	6 [110–115]
North America	21 [19, 116–135]
Mixed population from 2 continents	2 [136, 137]

Then the studies were assessed for year-wise distribution (Table 2). The maximum number of articles included were post-2010, and a maximum number of papers were obtained from the range 2016–2020.

**Table 2:** Year-wise distribution of the number of studies included in the study

Year	No. of studies
1995–2000	2
2001–2005	10
2006–2010	10
2011–2015	25
2016–2020	44
2021–2024	26

Nine studies [33, 39, 44, 50, 51, 54, 109, 121, 132] were included for objective 2 where infertility conditions were considered to estimate CAs among infertile and fertile couples who underwent ART. Table 3 describes the percentage of CAs conceived by infertile vs. fertile couples.

**Table 3:** Percentages of congenital anomalies found in infertile vs fertile couple in ART

Sl. No	Reference	Infertile male (%)	Fertile male (%)	Infertile female (%)	Fertile female (%)
1	Chen, Linjun et al. [33]	0.8	0.6		
2	Aliani, Fatemeh et al. [39]	0.53		0.59	
3	Hu, Shiqiao et al. [44]	2	1.6		
4	Hu, Shiqiao et al. [50]			1.1	1
5	Zhou, Wen-Jun et al. [51]	3.8	3.75		
6	Jwa, Seung Chik et al. [54]	1.1	1		
7	Oldereid, Nan B et al. [109]	2.3			
8	Wen, Shi Wu et al. [121]	0.79	1.37		
9	Xiong, Xu et al. [132]	3.1	2.6		

### ROB or quality assessment

ROB assessment was done using a checklist for case–control studies issued by JBI critical appraisal tools [20]. The studies were subjected to quality assessment by this checklist and 113 studies satisfied the assessment (Supplementary Information IV).

### Meta-analysis

#### Primary outcome: Incidence of congenital defects in ART vs. SC children

A total of 70 studies were included in this meta-analysis to assess the incidence of CAs in ART conceptions as compared to SC using the log risk ratio as the primary outcome measure.

**Fig. 2:**
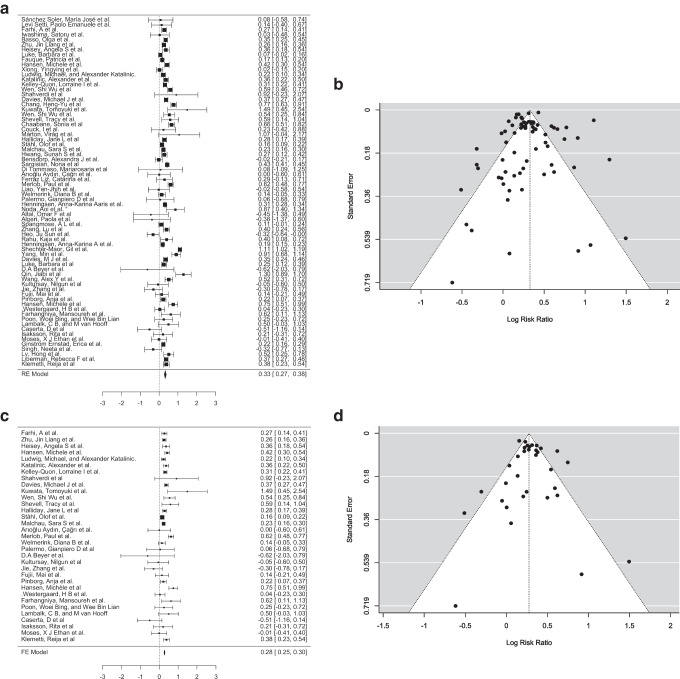

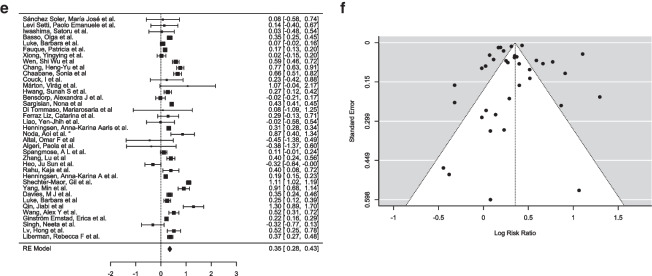
**a** Forest plot analysis comparing incidence of congenital anomalies in ART group (experimental group) vs spontaneous conception group (control group). **b** Funnel plot depicting the outliers of the meta-analysis which included 70 articles. **c** Forest plot analysis comparing incidence of congenital anomalies in ART group (experimental group) vs. spontaneous conception group (control group) (pre-2015). **d** Funnel plot depicting the outliers of the meta-analysis which included 33 articles. **e** Forest plot analysis comparing incidence of congenital anomalies in ART group (experimental group) vs. spontaneous conception group (control group) (after 2015). **f** Funnel plot depicting the outliers of the meta-analysis which included 37 articles

A random-effects model was applied to account for variability across studies, with the DerSimonian-Laird estimator used to calculate the between-study variance (τ^2^). The pooled estimate of the log risk ratio was μ = 0.327 (95% CI [0.274, 0.381]), indicating a statistically significant positive effect (Z = 12.0, *p* < 0.001) (Fig. 2a). The log risk ratios observed in individual studies ranged from − 0.618 to 1.494, with 84% of studies reporting a slightly higher incidence of CAs in the ART group compared to the SC group.

Heterogeneity among studies was substantial, as evidenced by Q (69) = 916.815 (*p* < 0.001), τ^2^ = 0.032 (SE = 0.0163), and I^2^ = 92.47%. These statistics indicate that 92.47% of the total variability in effect sizes can be attributed to true heterogeneity rather than sampling error. To further contextualize this variability, a 95% prediction interval was calculated, ranging from − 0.027 to 0.682. This range suggests that while the overall effect is positive, individual studies may yield negative outcomes in specific circumstances. This level of heterogeneity underscores the need to explore potential moderating factors or differences in study design, population characteristics, or intervention implementation.

Outlier and influence diagnostics were conducted to identify studies that may disproportionately affect the results. Two studies [60, 129] were identified as potential outliers, with studentized residuals exceeding the Bonferroni-corrected threshold of ± 3.384. Additionally, Cook’s distances flagged one study [129] as overly influential, indicating that its exclusion might impact the pooled effect size. These findings highlight the importance of considering the influence of specific studies in interpreting the meta-analysis results.

Publication bias was assessed using multiple methods. Neither the rank correlation test (*p* = 0.578) nor the regression test (*p* = 0.164) detected funnel plot asymmetry, suggesting minimal evidence of publication bias (Fig. 2b). A fail-safe analysis, conducted using Rosenthal’s approach, revealed a robust fail-safe *N* = 34,200 (*p* < 0.001), indicating that 34,200 null-result studies would be required to render the pooled effect size non-significant. This further supports the reliability of the observed effect.

The four studies excluded from the meta-analysis also showed a higher incidence of CAs in ART children compared to SC children [24–27].

In summary, the meta-analysis demonstrates a statistically significant increase in CAs in the ART group over the SC group. However, the high level of heterogeneity highlights substantial variability in outcomes across studies, suggesting that the effectiveness of ART may depend on contextual or population-specific factors. Outliers and influential studies were identified but did not compromise the robustness of the overall findings. The lack of significant publication bias further strengthens the validity of the results. Future research should explore moderating variables and refine the intervention to optimize its effectiveness across diverse settings.

#### A comparative analysis of studies done pre- and post-2015 to evaluate the incidence of congenital anomalies in ART children

To find the differences in the incidence of CAs in children born through ART vs. SC, we set the timeline as 2015 to analyze the differences (if any) before and after that year. We sought to find differences caused by advancements in ART and its impact on neonatal health.

##### Pre-2015

A total of 33 studies published before 2015 were included in this meta-analysis to assess and evaluate the incidence of CAs in children born after ART compared to spontaneously conceived children.

A random-effects model was applied to account for potential variability between studies, with the DerSimonian-Laird estimator used to calculate the between-study variance (τ^2^ = 0.0149, SE = 0.0074). The estimated log risk ratio was μ = 0.2985 (95% CI: 0.2378 to 0.3593), indicating a statistically significant positive effect (*z* = 9.6375, *p* < 0.0001), suggesting that the ART group had slightly higher chances of anomalies than the SC group (Fig. 2c). Observed log risk ratios in individual studies ranged from − 0.6183 to 1.4945, with 85% of studies reporting positive effects.

Heterogeneity analysis revealed a moderate level of variability across studies, with (Q (32) = 104.8409, τ^2^ = 0.0149, *p* < 0.0001, and I^2^ = 69.4776%, suggesting that approximately more than two-thirds of the observed variation in effect sizes is due to differences in true effects rather than random error. This level of heterogeneity suggests that study-level characteristics, such as population demographics, clinical protocols, or contextual differences, may influence the outcomes and require further exploration.

Outlier and influence diagnostics were conducted to identify studies that could disproportionately affect the pooled results. No studies were flagged as outliers based on studentized residuals, as none exceeded the Bonferroni-corrected threshold of ± 3.144. However, Cook’s distances identified one study [45] as overly influential, suggesting that its exclusion might alter the pooled effect size. These findings emphasize the need to carefully interpret the influence of specific studies and consider sensitivity analyses to confirm the robustness of the results.

Publication bias was assessed using both rank correlation and regression tests for funnel plot asymmetry. The rank correlation test did not show significant evidence of asymmetry (*p* = 0.6781), but the regression test indicated significant funnel plot asymmetry (*p* = 0.5261), suggesting possible publication bias (Fig. 2d). A fail-safe *N* analysis, calculated using Rosenthal’s approach, reported a fail-safe *N* = 3410 (*p* < 0.001), indicating that 3410 unpublished null-result studies would be needed to nullify the observed pooled effect size. While the fail-safe N indicates robustness, the evidence of funnel plot asymmetry highlights potential biases in the literature, such as selective reporting of positive outcomes.

##### Post-2015

A total of 37 studies published after 2015 were included in this meta-analysis to assess and evaluate the incidence of CAs in children born after ART compared to spontaneously conceived children.

A random-effects model was applied to account for potential variability between studies, with the DerSimonian-Laird estimator used to calculate the between-study variance (τ^2^ = 0.0374, SE = 0.0237). The estimated log risk ratio was μ = 0.3532 (95% CI: 0.2766 to 0.4299), indicating a statistically significant positive effect (*z* = 9.0326, *p* < 0.0001), suggesting that ART had slightly higher chances of anomalies than SC (Fig. 2e). Observed log risk ratios in individual studies ranged from − 0.445 to 1.296, with 95% of studies reporting positive effects.

Heterogeneity analysis revealed a moderate level of variability across studies, with Q (36) = 794.6589, τ^2^ = 0.0374, *p* < 0.0001, and I^2^ = 95.4698%, suggesting that observed variation in effect sizes is due to differences in true effects. This level of heterogeneity suggests that study-level characteristics, such as population demographics, clinical protocols, or contextual differences, may influence the outcomes and merit further exploration.

Outlier and influence diagnostics were conducted to identify studies that could disproportionately affect the pooled results. Two studies were flagged as outliers based on studentized residuals, as they exceeded the Bonferroni-corrected threshold of ± 3.205 [60, 129]. However, Cook’s distances identified one study as overly influential, suggesting that its exclusion might alter the pooled effect size [129]. These findings emphasize the need to carefully interpret the influence of specific studies and consider sensitivity analyses to confirm the robustness of the results.

Publication bias was assessed using both rank correlation and regression tests for funnel plot asymmetry (Fig. 2f). Neither of these tests showed significant evidence of asymmetry (*p* = 0.825 and *p* = 0.227, respectively). A fail-safe *N* analysis, calculated using Rosenthal’s approach, reported a fail-safe *N* = 15,953 (*p* < 0.001), indicating that 15,953 unpublished null-result studies would be needed to nullify the observed pooled effect size. While the fail-safe *N* indicates robustness, the possibility of selective reporting of positive results cannot be ruled out entirely.

To conclude, this sub-analysis revealed that there were no significant changes observed in the outcome with respect to the timeline employed. The results were consistent and showed slightly higher anomalies in children born via ART vs. SC. Future research should explore the factors contributing to heterogeneity, including study design differences and population-specific characteristics, to better understand the reason. These considerations will help refine the intervention and enhance its generalizability across diverse clinical settings.

### Secondary outcomes

#### Incidence of congenital anomalies in children born after IVF vs. ICSI

**Fig. 3:**
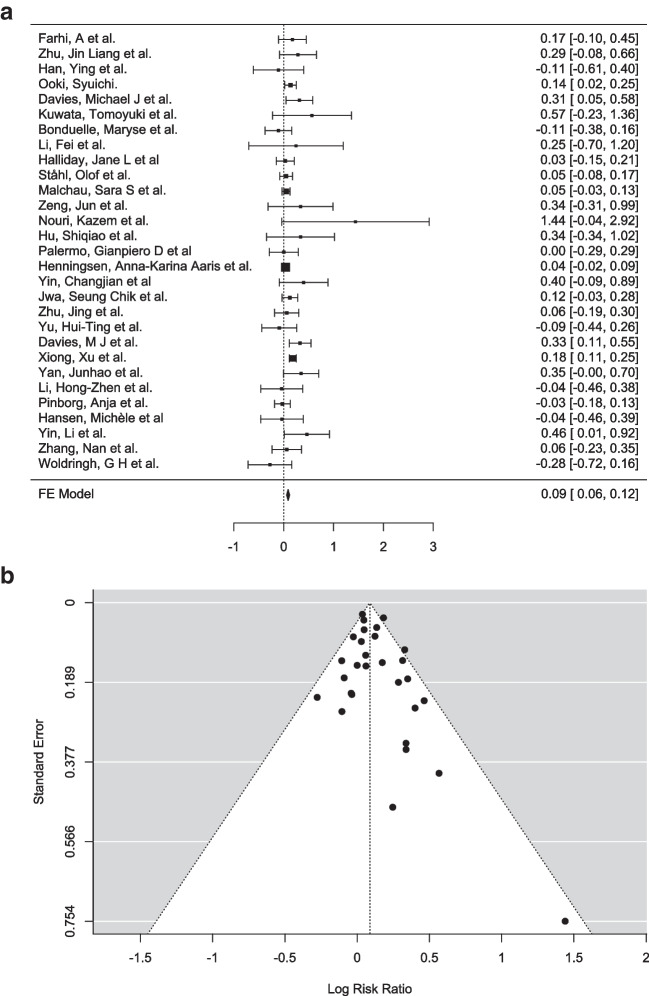
**a** Forest plot analysis comparing incidence of congenital anomalies in ICSI group (experimental group) vs. IVF group (control group). **b** Funnel plot depicting the outliers of the meta-analysis including 29 studies

A meta-analysis was conducted to evaluate the incidence of CAs conceived through different ART methods, IVF and ICSI. A total of 29 studies were included in this analysis. The fixed effects model yielded a log risk ratio of 0.0877 (95% CI: 0.058 to 0.118, *Z* = 5.72, *p* < 0.001), indicating a statistically significant positive association, as shown in the figure below (Fig. 3a). This suggests that ICSI had more effect on outcome than IVF. The 95% confidence interval for the log risk ratio ranged from − 0.277 to 1.440, with 76% of studies reporting positive estimates. These results indicated a statistically significant lower risk of congenital abnormalities for IVF to ICSI, with the negative log risk ratio signifying the reduced risk associated with the IVF group.

The heterogeneity was modest, with I^2^ = 35.31%, Q (28) = 43.285, *p* = 0.033, suggesting that approximately one-third of the variability in effect sizes across studies was due to true differences rather than sampling error. Cook’s distances identified three studies [81, 132, 138] as overly influential due to their relatively large weights compared to the rest of the studies. However, evaluation of the studentized residuals showed there was no study which exceeded the value ± 3.1340 and therefore there was no proof of any outliers in the context of this model. Publication bias was assessed using funnel plot asymmetry tests, including the rank correlation and regression tests, neither of which provided evidence of asymmetry (rank correlation: *p* = 0.211; regression: *p* = 0.095) (Fig. 3b). The fail-safe *N* analysis calculated a value of 283 (*p* < 0.001), indicating that 283 null result studies would be required to nullify this effect indicating the robustness of this analysis.

Overall, this meta-analysis revealed a modest but significant pooled effect size favouring IVF over ICSI with minimal evidence of publication bias and no extreme outliers identified. However, the presence of heterogeneity and influential studies warrants further investigation into study-level characteristics that may explain the observed variability.

#### Children with congenital anomalies born after fresh ET vs. FET

**Fig. 4:**
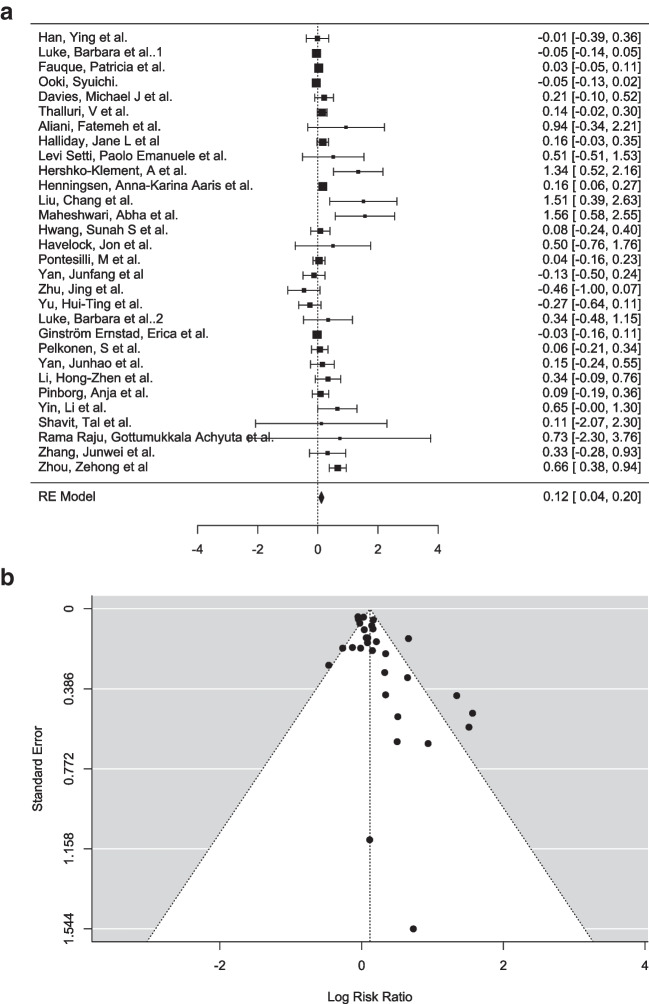
**a** Forest plot analysis comparing incidence of congenital anomalies in fresh ET group (experimental group) vs. FET group (control group). **b** Funnel plot depicting the outliers of the meta-analysis including 30 studies

A total of 30 studies were included in this meta-analysis to assess the efficacy of fresh embryo transfer (ET) compared to frozen embryo transfer (FET) using the log risk ratio as the primary outcome measure. A random-effects model was applied to account for potential variability between studies, with the DerSimonian-Laird estimator used to calculate the between-study variance (τ^2^ = 0.0199, SE = 0.0121). The pooled estimate of the log risk ratio was μ = 0.117 (95% CI [0.036, 0.199]), indicating a statistically significant positive effect (*Z* = 2.84, *p* = 0.005), suggesting that fresh ET has a slightly increased effect on congenital anomalies than FET (Fig. 4a). Observed log risk ratios in individual studies ranged from − 0.465 to 1.565, with 77% of studies reporting positive effects. Despite the overall positive outcome, the prediction interval (− 0.171 to 0.406) reveals that some studies may yield negative results under specific circumstances, highlighting potential variability in intervention effectiveness.

Heterogeneity analysis revealed a moderate level of variability across studies, with Q (29) = 80.772, *p* < 0.001, and I^2^ = 64.1%, suggesting that approximately two-thirds of the observed variation in effect sizes is due to differences in true effects rather than random error. The H^2^ statistic (2.785) further supports this finding, indicating that the total variability in observed effects exceeds the expected variability from sampling error. This level of heterogeneity suggests that study-level characteristics, such as population demographics, clinical protocols, or contextual differences, may influence the outcomes and require further exploration.

Outlier and influence diagnostics were conducted to identify studies that could disproportionately affect the pooled results. No studies were flagged as outliers based on studentized residuals, as none exceeded the Bonferroni-corrected threshold of ± 3.144. However, Cook’s distances identified one study [57] as overly influential, suggesting that its exclusion might alter the pooled effect size. These findings emphasize the need to carefully interpret the influence of specific studies and consider sensitivity analyses to confirm the robustness of the results.

Publication bias was assessed using both rank correlation and regression tests for funnel plot asymmetry. The rank correlation test did not show significant evidence of asymmetry (*p* = 0.058), but the regression test indicated significant funnel plot asymmetry (*p* < 0.001), suggesting possible publication bias (Fig. 4b). A fail-safe *N* analysis, calculated using Rosenthal’s approach, reported a fail-safe *N* = 247 (*p* < 0.001), indicating that 247 unpublished null-result studies would be needed to nullify the observed pooled effect size. While the fail-safe *N* indicates robustness, the evidence of funnel plot asymmetry highlights potential biases in the literature, such as selective reporting of positive outcomes.

In conclusion, this meta-analysis demonstrates a statistically significant pooled effect indicating that fresh ET has an increased effect on anomalies in comparison to FET, with a moderate level of heterogeneity (I^2^ = 64.1%) indicating variability in study outcomes. Although no extreme outliers were detected, the identification of an influential study and evidence of potential publication bias suggest that the findings should be interpreted cautiously. Future research should explore the factors contributing to heterogeneity, including study design differences and population-specific characteristics, to better understand the contexts in which ET is most effective. These considerations will help refine the intervention and enhance its generalizability across diverse clinical settings.

####  Children with congenital anomalies born after blastocyst transfer vs. cleavage transfer

**Fig. 5:**
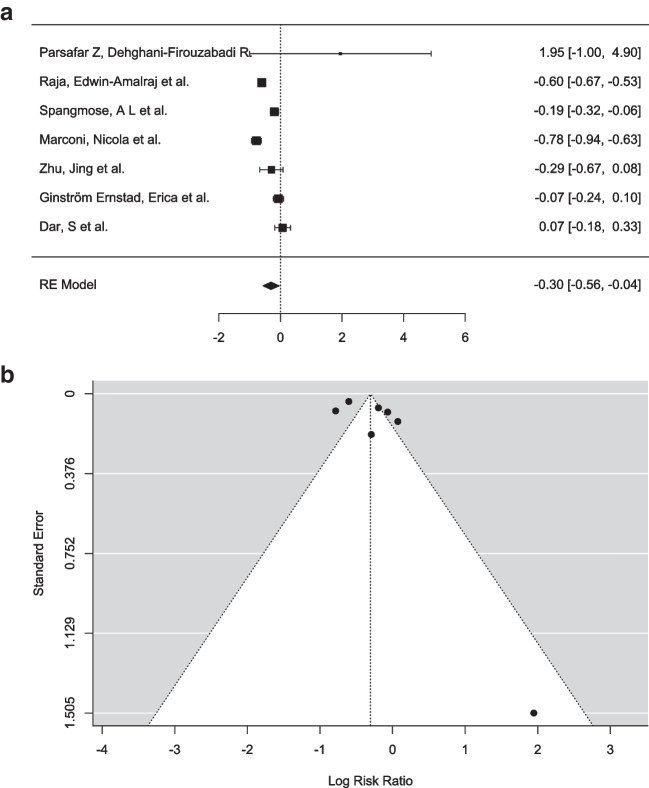
**a** Forest plot analysis comparing incidence of congenital anomalies in blastocyst transfer group (experimental group) vs. cleavage transfer group (control group). **b** Funnel plot depicting the outliers of the meta-analysis including 7 studies

A meta-analysis was conducted to evaluate the efficacy of blastocyst-stage embryo transfer (Day 5) compared to cleavage-stage embryo transfer (Day 3) in ART. Seven studies were included, with log risk ratios ranging from − 0.784 to 1.946, and a majority (71%) reporting negative estimates. A random-effects model was applied, using the DerSimonian-Laird estimator to account for between-study variance (τ^2^ = 0.0952, SE = 0.0805). The pooled log risk ratio was μ = − 0.304 (95% CI [− 0.564, − 0.044], *Z* = − 2.29, *p* = 0.022), indicating a statistically significant negative effect favoring blastocyst-stage transfer (Fig. 5a). This suggests that, on average, blastocyst transfer is associated with a lesser incidence of anomalies compared to cleavage-stage transfer. However, the prediction interval (− 0.962 to 0.354) highlights that individual studies may show a positive effect under specific conditions.

Heterogeneity across studies was substantial, with Q (6) = 88.411, *p* < 0.001, I^2^ = 93.21%, and H^2^ = 14.735, suggesting that 93.21% of the observed variability was due to true differences across studies rather than random error. This high degree of heterogeneity underscores the need to examine potential moderating factors, such as population characteristics, study design, or clinical protocols. Outlier analysis, based on studentized residuals, revealed no studies exceeding the Bonferroni-corrected threshold of ± 2.690, and Cook’s distances identified no studies as overly influential. These findings suggest that the results were robust to the influence of individual studies.

Publication bias was assessed through funnel plot asymmetry tests, including the rank correlation and regression tests (Fig. 5b). Neither the rank correlation test (*p* = 0.773) nor the regression test (*p* = 0.082) indicated significant asymmetry. Additionally, a fail-safe *N* = 320 (*p* < 0.001) was calculated using Rosenthal’s approach, indicating that 320 null-result studies would be required to render the observed effect non-significant. This supports the reliability of the results despite the limited number of included studies.

In conclusion, the meta-analysis demonstrates a statistically significant pooled negative effect, which suggested a slightly reduced risk with blastocyst-stage embryo transfer. However, the substantial heterogeneity across studies (I^2^ = 93.21%) and the prediction interval suggest that the results may not be consistent across all contexts. No evidence of publication bias was detected, and the findings appeared robust to the influence of individual studies. Further research is warranted to explore the factors contributing to variability in outcomes and to better understand the conditions under which blastocyst transfer might achieve optimal efficacy. These results have important implications for clinical decision-making in assisted reproductive technology, highlighting the need for tailored approaches to embryo transfer based on patient-specific and contextual factors.

The meta-analyses showed a different profile for the risk of congenital abnormalities connected to distinct ART methods. Reduced risk was noted when IVF was used instead of ICSI, FET was used instead of fresh ET, and blastocyst-stage transfer was used instead of cleavage-stage transfer. These results are useful for understanding the risks and benefits of various ART interventions to make better informed clinical decisions in ART applications and to improve future studies of reproductive healthcare.

## Discussion

### General interpretations

The result of the current systematic review and meta-analysis revealed a statistically significant increase in the incidence of CAs in children born after ART compared with children born after SC Our study included 117 studies (3 of them from the Indian cohort) in which 70 studies were chosen for meta-analysis for comparison of anomalies in ART vs. SC group. This included 768,929 children in the ART group and 40,709,337 children in the SC group. This provided a comprehensive robust result. This analysis revealed an average log risk ratio of 0.328, translating to an approximately 39% increase in the risk of CAs for ART-conceived children. This aligns with previous meta-analyses [14, 139, 140] which had a similar result as ours. While this increase in relative risk sounds substantial, the absolute risk difference must be placed in context. The incidence of CAs was set around 2.48% for SC children in the included studies; thus, the estimated incidence for ART-conceived children, when applying this 39% relative increase observed in our analysis, is 3.45%, resulting in an absolute risk difference of 0.97 percentage points (approximately 1%).

Thus, while there is a slight elevation in risk with ART, the absolute increase is clinically modest. Such information should be communicated to physicians and potential parents effectively so that they underscore the importance of deeper understanding and informed decision making when it comes to ART. A recent meta-analysis on the same topic also suggested the same but just for major anomalies [141]. Our study covered a more comprehensive analysis due to the higher number of studies included and more subgroup analysis (ET vs. FET, day 5 vs. day 3 transfer) which shed insights on the types of ART treatment and their potential effect.

Our analysis identified variability in risk depending on the specific ART technique used. For instance, children conceived through IVF showed a lower risk of CAs compared to those conceived via ICSI, while Day 5 transfer and FET showed lower risk compared to that of day 3 transfer and fresh ET.

### Limitations

There are some strengths and limitations associated with the reviewed source regarding the identification of the evidence. The strengths could be ascribed to the permissiveness of the papers encompassing large heterogeneity revealed by high I^2^ values declared in meta-analysis. However, the increased risk of CAs are not solely from ART but they could also originate from pre-existing and uncontrollable factors such as maternal age, lifestyle factors, fertility issues, genetics, and other health conditions. Studies like these help in deeper understanding of the origin of these anomalies and the role ART might have in it.

- Advanced maternal age: One of the more significant confounders for CAs may include advanced maternal age [98, 114]. As maternal age increases, chromosomal abnormalities and other anomalies rise. This is often confounded by selection for ART patients, as many ART patients tend to be older and might therefore be suffering from infertility.
- Infertility and subfertility: Couples initiating ART may have a pre-diagnosed fertility disorder in either or both male and female partners linked with several associated medical conditions, and these preconditions have contributed to increasing CA disposition risk. Infertility can arise in women with, among countless other possible causes, advanced maternal age, polycystic ovary syndrome (PCOS), or endometriosis.
- Health conditions: Many ART patients may have pre-existing health conditions such as diabetes, hypertension, thyroid disorders, or obesity. These are known to increase the risk of adverse pregnancy outcomes. For example, maternal diabetes is associated with a higher risk of neural tube defects and other CAs [142]. Conditions like these may confound the relationship between ART and congenital anomalies.
- Lifestyle factors: Factors such as smoking, alcohol consumption, diet, and physical activity may also be implicated in the risk of CAs. While these factors are important to consider within any population, they may, nevertheless, be more prevalent or exert different effects on ART treatments.
- Genetic predispositions and family history: The genetic factors have a significant role in affecting the offspring’s health which may get transmitted independently of ART intervention. In addition, some ART treatments involve genetic screening and preimplantation genetic testing, which could reveal genetic abnormalities that might be missed otherwise in natural pregnancies. Such variations have the net effect of making accounting for these variables less routine, which influences the overall quality of the risk estimates that are tied to ART.

Moreover, mainly because of the type of studies in the sample, the existence of outliers within the sample or influential studies poses a great deal of confusion in the results displayed. First, given the logistic nature of the models utilized, the relationship between the covariates and time-to-event outcomes may be non-linear and non-proportional, which could be factors for confounding in case of imbalance in the covariate distribution between the treatment groups, though balanced randomization was employed in the present study [143, 144].

It should also be pointed out that only studies reported in the English language have been included in the current analysis. Such a language limitation might exclude some relevant data from such research works that are in languages other than English. Therefore, it introduces a limitation of language bias, and this reaffirms the fact that the results cannot be generalized.

Another limitation connected with the systematic review is the potential of reporting bias because only studies that have been published have been included. No publication bias of the primary outcome is identified when considering the funnel plot; nevertheless, it can be envisaged that many similar studies were not published at all and, therefore, affected the outlook of the results.

### Implications of the results and future directions

Therefore, the findings of this review are helpful to clinicians, policymakers, and researchers who are concerned about prospective parents and fulfilling their natural desire for offspring and improving their patients’ quality of life. In understanding the study conducted above, clinicians and reproductive specialists should note that birth through ART exposes a newborn to anomalies more than natural conception. It is necessary to present such information specifically for the decision-making referring to the choice of ART procedures. It is to be emphasized that each couple should think about the possible risks and benefits of their future reproductive decisions in order to make a conscious and well-informed decision.

In the practice of ART, timing of embryo transfer is critical to the success and safety of the treatment. As to the comparative risks of embryo transfer on day 3 as opposed to day 5, very little research has been done. The present review emphasizes the need for further studies to see how timing of transfer may influence the subsequent incidence of CAs. Since embryos at different stages may possess different implantation and developmental capabilities, these variations could affect their long-term outcomes. Future studies should be conducted to compare these transfer strategies while controlling for confounding factors to see if the specific timing of transfer leads to a lower risk of CAs in the long term.

Another important finding of this review is the marginally increased risk of CAs in children conceived with fresh ETs compared to those conceived through FETs [64, 128]. While there have been quite favorable outcomes from FETs, fresh ETs are still being conducted. The reasons for this increased risk are unclear, but they may include the hormonal environment during the cycle in which fresh ET takes place, embryo stage at the time of transfer, or maternal–fetal interactions [145]. Thus, it will be of great importance for future studies to investigate the physiological differences between these two transfer methods, as knowledge of these factors could allow for optimization of ART procedures in an attempt to reduce risks.

Apart from timing and technique of embryo transfer, other ART-related factors such as hormonal treatment for uterine preparation or use of PGD should also be given due consideration. Hormonal treatment, in particular, can have long-lasting effects on embryonic and fetal development. Moreover, socio-economic and lifestyle factors must be considered, along with maternal health, to enable such research to be conducted.

To address the limitations of our study, studies should focus on more confounding factors to find the root cause of CAs. Along with this, establishing a longitudinal follow-up of ART-conceived children is crucial in establishing the entire spectrum of health risks associated with ART treatment, including CAs, developmental disorders, and long-term health effects. Larger scale and methodologically better-designed cohort studies should be done involving children born through ART compared with those born without ART usage and compared while statistically controlling for such potential confounders as parents’ age, size, and socio-economic status. In addition, future studies should also focus on providing detailed information on the effect of various other ART interventions such as hormonal treatments, preimplantation genetic testing, culture conditions and other practices on the incidence of CAs in ART-conceived children to bring refinements in ART.

## Conclusion

To summarize, this systematic review and meta-analysis aimed to offer a detailed understanding of the study objective which was to examine the odds of CAs in ART children as compared to children conceived naturally. The preliminary results of this research show that ART-conceived offspring have a slightly higher rate of birth defects.

The secondary analysis builds upon this discussion by reemphasizing the fact that implementing different ART methods entails disparate risks for children and families. Most of the studies with matched samples were therefore informative; it was noted that the meta-analysis of the current comparative studies of IVF and ICSI indicated a statistically significant lower risk of CAs associated with IVF than with ICSI. Fresh ET had a slightly higher incidence of anomalies in children as compared to FET. The analysis also revealed that cleavage-stage transfer posed a slightly higher risk of CAs as compared to blastocyst transfer. Furthermore, amid other perceived factors, the analysis of the study and use of underlying causes of infertility among parents establish that CA risk also depends on parental factors. This calls for the need to consider the parents’ characteristics as well as the specific infertility factors when evaluating the possibility of the increased risk for CAs. The results are summarized and tabulated in Table 4.

**Table 4:** Summary of risk estimates for primary and secondary outcomes in ART

Outcomes	Log risk ratio	Absolute risk	Interpretation
ART vs. SC (control)	Total: 0.327/1.39 (39% increase)	SC: 2.48% vs. ART: 3.45% (↑ 0.97%)	ART is found to have slightly higher risk as compared to SC
	Pre-2015: 0.298/1.35 (35% increase)	SC: 3.1% vs. ART: 4.19% (↑ 1.09%)	
	Post-2015: 0.353/1.42 (42% increase)	SC: 2.41% vs. ART: 3.42% (↑ 1.01%)	
IVF vs. ICSI (control)	0.087/1.09 (9% increase)	IVF: 3.06% vs. ICSI: 3.34% (↑ 0.28%)	ICSI shows marginally higher risk than IVF
Fresh ET vs. FET(control)	0.117/1.12 (12% increase)	FET: 1.97% vs. Fresh ET: 2.21% (↑ 0.24%)	Fresh ET shows slightly higher risk
Day 5 vs. Day 3 Transfer (control)	− 0.304/0.76 (26% decrease)	Day 3: 4.10% vs. Day 5: 3.03% (↓ 1.07%)	Day 3 transfer shows slightly higher risk

Therefore, as affirmed by this systematic review and meta-analysis, there is importance in carrying out regular follow-up of the health of children conceived through ART. The finding of this study should help enlighten clinical practices, by offering directions on how the ART should be practiced, together with the counseling that should accompany it in prospective parentage. Although the available options for the treatment of infertility continue to evolve, it is crucial to align the positive impact of ART with the identification of adverse outcomes for the sake of future generations.

## Supplementary Information

Below is the link to the electronic supplementary material.

Supplementary file1 (DOCX 31 KB)

Supplementary file2 (DOCX 12 KB)

Supplementary file3 (XLSX 125 KB)

Supplementary file4 (XLSX 63 KB)

## Data Availability

Not applicable as no new data were created in the study as it is a systematic review and meta-analysis. All data analyses are included in the manuscript and supplementary information. This review was registered in PROSPERO on 20/05/2024 (ID: CRD42024548861), as mentioned in the “Materials and methods” section.
