# Supplementary material for: Congenital anomalies observed in children conceived through assisted reproductive technology—a systematic review and meta-analysis
Source: J Assist Reprod Genet. 2025 Mar 31;42(5):1547–65. doi: 10.1007/s10815-025-03454-0 (PMC12167209; doi:10.1007/s10815-025-03454-0)
Supplement: Supplementary file 2 — Supplementary file2 (DOCX 12 KB) [file 10815_2025_3454_MOESM2_ESM.docx]

**Supplementary Information II**

| **Search engine** | **Search strategy** | **Papers obtained** |
| --- | --- | --- |
| **PubMed**  [Link](https://pubmed.ncbi.nlm.nih.gov/) | (("Congenital Abnormalities"[MeSH Terms] OR "congenital malformation*"[All Fields]) AND ("case reports"[Publication Type] OR "clinical study"[Publication Type] OR "clinical trial"[Publication Type] OR "clinical trial protocol"[Publication Type] OR "clinical trial, phase i"[Publication Type] OR "clinical trial, phase ii"[Publication Type] OR "clinical trial, phase iii"[Publication Type] OR "clinical trial, phase iv"[Publication Type] OR "controlled clinical trial"[Publication Type] OR "evaluation study"[Publication Type] OR "meta-analysis"[Publication Type] OR "multicenter study"[Publication Type] OR "observational study"[Publication Type] OR "randomized controlled trial"[Publication Type] OR "review"[Publication Type] OR "systematic review"[Filter] OR "twin study"[Publication Type]) AND (("reproductive techniques, assisted"[MeSH Terms] OR "artificial reproductive technology*"[All Fields] OR "IVF/ICSI"[All Fields]) AND ("case reports"[Publication Type] OR "clinical study"[Publication Type] OR "clinical trial"[Publication Type] OR "clinical trial protocol"[Publication Type] OR "clinical trial, phase i"[Publication Type] OR "clinical trial, phase ii"[Publication Type] OR "clinical trial, phase iii"[Publication Type] OR "clinical trial, phase iv"[Publication Type] OR "controlled clinical trial"[Publication Type] OR "evaluation study"[Publication Type] OR "meta-analysis"[Publication Type] OR "multicenter study"[Publication Type] OR "observational study"[Publication Type] OR "randomized controlled trial"[Publication Type] OR "review"[Publication Type] OR "systematic review"[Filter] OR "twin study"[Publication Type])) AND (("case reports"[Publication Type] OR "classical article"[Publication Type] OR "clinical study"[Publication Type] OR "clinical trial"[Publication Type] OR "clinical trial, phase i"[Publication Type] OR "clinical trial, phase ii"[Publication Type] OR "clinical trial, phase iii"[Publication Type] OR "clinical trial, phase iv"[Publication Type] OR "controlled clinical trial"[Publication Type] OR "evaluation study"[Publication Type] OR "multicenter study"[Publication Type] OR "observational study"[Publication Type] OR "randomized controlled trial"[Publication Type] OR "twin study"[Publication Type]) AND "loattrfull text"[Filter] AND "humans"[MeSH Terms] AND "english"[Language])) AND ((casereports[Filter] OR classicalarticle[Filter] OR clinicalstudy[Filter] OR clinicaltrial[Filter] OR clinicaltrialphasei[Filter] OR clinicaltrialphaseii[Filter] OR clinicaltrialphaseiii[Filter] OR clinicaltrialphaseiv[Filter] OR comparativestudy[Filter] OR controlledclinicaltrial[Filter] OR evaluationstudy[Filter] OR multicenterstudy[Filter] OR observationalstudy[Filter] OR randomizedcontrolledtrial[Filter] OR twinstudy[Filter]) AND (fft[Filter]) AND (humans[Filter]) AND (english[Filter]) AND (allchild[Filter] OR newborn[Filter] OR allinfant[Filter] OR infant[Filter] OR preschoolchild[Filter] OR child[Filter] OR adolescent[Filter] OR alladult[Filter] OR youngadult[Filter] OR adult[Filter] OR middleagedaged[Filter] OR middleaged[Filter])) | **480** |
| **Embase**  [Link](https://www.embase.com/search/quick) | title: ('congenital abnormality'/exp OR 'congenital abnormality' OR 'congenital defect'/exp OR 'congenital defect' OR 'birth defect'/exp OR 'birth defect' OR 'congenital malformation'/exp OR 'congenital malformation' OR 'fetal malformation'/exp OR 'fetal malformation' OR 'congenital anomaly'/exp OR 'congenital anomaly' OR 'fetal abnormality':ti) AND ([article]/lim OR [data papers]/lim) AND [english]/lim AND ([embryo]/lim OR [fetus]/lim OR [newborn]/lim OR [infant]/lim OR [child]/lim OR [preschool]/lim OR [school]/lim OR [adolescent]/lim OR [adult]/lim OR [young adult]/lim OR [middle aged]/lim) AND [humans]/lim AND [abstracts]/lim AND [clinical study]/lim  441,862  Title: ('assisted reproductive technique' OR 'assisted reproductive technology' OR 'reproductive technology' OR 'artificial reproductive technology' OR 'ivf/icsi':ti) AND ([article]/lim OR [data papers]/lim) AND [english]/lim AND ([embryo]/lim OR [fetus]/lim OR [newborn]/lim OR [infant]/lim OR [child]/lim OR [preschool]/lim OR [school]/lim OR [adolescent]/lim OR [adult]/lim OR [young adult]/lim OR [middle aged]/lim) AND [humans]/lim AND [abstracts]/lim AND [clinical study]/lim  5,108  abstract: ('congenital abnormality' OR 'congenital defect' OR 'birth defect' OR 'congenital malformation' OR 'fetal malformation' OR 'congenital anomaly' OR 'fetal abnormality':ab) AND ([article]/lim OR [data papers]/lim) AND [english]/lim AND ([embryo]/lim OR [fetus]/lim OR [newborn]/lim OR [infant]/lim OR [child]/lim OR [preschool]/lim OR [school]/lim OR [adolescent]/lim OR [adult]/lim OR [young adult]/lim OR [middle aged]/lim) AND [humans]/lim AND [abstracts]/lim AND [clinical study]/lim  30,047  ('assisted reproductive technique' OR 'assisted reproductive technology' OR 'reproductive technology' OR 'artificial reproductive technology' OR 'ivf/icsi':ab) AND ([article]/lim OR [data papers]/lim) AND [english]/lim AND ([embryo]/lim OR [fetus]/lim OR [newborn]/lim OR [infant]/lim OR [child]/lim OR [preschool]/lim OR [school]/lim OR [adolescent]/lim OR [adult]/lim OR [young adult]/lim OR [middle aged]/lim) AND [humans]/lim AND [abstracts]/lim AND [clinical study]/lim  6,231 | **575** |
| **WOS**  [Link](https://www.webofscience.com/wos/woscc/advanced-search) | TI=('congenital abnormality' OR 'congenital defect' OR 'birth defect' OR 'congenital malformation' OR 'fetal malformation' OR 'congenital anomaly' OR 'fetal abnormality') and Article or Data Paper (Document Types) and English (Languages) 11,597  TI=('assisted reproductive technique' OR 'assisted reproductive technology' OR 'reproductive technology' OR 'artificial reproductive technology' OR 'ivf/icsi') and Article (Document Types) and English (Languages) 3,550  AB=('congenital abnormality' OR 'congenital defect' OR 'birth defect' OR 'congenital malformation' OR 'fetal malformation' OR 'congenital anomaly' OR 'fetal abnormality') and English (Languages) and Article or Data Paper (Document Types) 91,295  AB=('assisted reproductive technique' OR 'assisted reproductive technology' OR 'reproductive technology' OR 'artificial reproductive technology' OR 'ivf/icsi') and English (Languages) and Data Paper or Article (Document Types) 15,945 | **556** |
| **Scopus**  [Link](https://www.scopus.com/search/form.uri?display=basic#basic) | ( 'congenital AND abnormality' OR 'congenital AND defect' OR 'birth AND defect' OR 'congenital AND malformation' OR 'fetal AND malformation' OR 'congenital AND anomaly' OR 'fetal AND abnormality' ) AND ( 'assisted AND reproductive AND technique' OR 'assisted AND reproductive AND technology' OR 'reproductive AND technology' OR 'reproductive AND technique' OR 'ivf/icsi' ) AND ( LIMIT-TO ( DOCTYPE , "ar" ) ) AND ( LIMIT-TO ( LANGUAGE , "English" ) ) | **72** |

**Congenital anomalies observed in children conceived through assisted reproductive technology - A systematic review and meta-analysis**

**Paripoorna Bhat^1^, Vijay Shree Dhyani^2^, Vani Lakshmi^3^, Shubhashree Uppangala^1^ , Satish Kumar Adiga^4^, Prashanth Adiga^5^, Pratap Kumar^5^,**

***Aditi Gupta^1^ (ORCID: 0000-0003-3647-8148)**

^1^Division of Reproductive Genetics, Department of Reproductive Science, Kasturba Medical College, Manipal, Manipal Academy of Higher Education, Manipal, Karnataka, India- 576104

^2^Kasturba Medical College, Manipal, Manipal Academy of Higher Education, Manipal, Karnataka, India- 576104

^3^Department of Data Science, Prasanna School of Public Health, Manipal, Manipal Academy of Higher Education, Manipal, Karnataka, India- 576104

^4^Centre of Excellence in Clinical Embryology, Department of Reproductive Science, Kasturba Medical College, Manipal, Manipal Academy of Higher Education, Manipal, Karnataka, India- 576104

^5^Department of Reproductive Medicine and Surgery, Kasturba Medical College, Manipal, Manipal Academy of Higher Education, Manipal, Karnataka, India- 576104

***Corresponding author-** [aditi.g@manipal.edu](mailto:aditi.g@manipal.edu); [aditiguptagenetics@gmail.com](mailto:aditiguptagenetics@gmail.com)

Submitted to Journal of Assisted Reproduction and Genetics.
